# “It turns out I was wrong”: a thematic analysis of medical students’ reflective writing in an e-learning course on communication skills

**DOI:** 10.1186/s12909-025-08172-w

**Published:** 2025-12-04

**Authors:** Laura Janssen, Kristina Schick, Sylvia Irene Donata Pittroff, Sabine Reiser, Johannes Bauer, Pascal O. Berberat, Martin Gartmeier

**Affiliations:** 1 TUM School of Medicine and Health, Department Clinical Medicine, TUM Medical Education Center, Technical University of Munich, TUM University Hospital, Munich, Germany; 2 Educational Research and Methodology, University of Erfurt, Erfurt, Germany; 3 Institute of Medical Education, Medical Faculty and University Hospital Carl Gustav Carus, TUD Dresden University of Technology, Dresden, Germany

**Keywords:** Reflection, E-learning, Medical education, Physician-patient communication, Clinical communication, Reflective writing, Reflective practice, Thematic analysis

## Abstract

**Background:**

Self-reflection and e-learning represent two recent shifts in medical education, offering benefits for teaching clinical communication, a core component of medical training. Integrating reflection exercises into e-learning environments can encourage students to engage deeply with course content and their own experiences, fostering self-awareness, novel insights, and professional growth. However, few studies have explored how online learning environments might support students in reflecting on their learning experiences. In particular, research is lacking on how medical students perceive their learning experiences in online environments, including reflection, especially in the context of clinical communication training. This study examines how students approached structured reflective writing within an e-learning course on physician-patient communication to explore (I) the learning content addressed in students’ reflections and (II) the insights students gained through the course.

**Methods:**

First-year clinical medical students (*n* = 55) participated in an e-learning course, which consisted of three modules: communication basics, consultation structuring, and empathy and emotions. Each module included three guided prompts to encourage reflection. All participants composed nine written reflections. Qualitative thematic analysis of all 495 reflective texts was conducted to identify themes and insights.

**Results:**

The students engaged with core communication topics and developed greater self-awareness regarding their communicative practice. The reflections revealed challenges, perceived skill gaps, and new understanding. Five core themes were identified: 1. patient-centred communication, 2. conversation structure, 3. emotions, 4. learning experiences, and 5. attitudes. Themes 1–3 primarily described specific learning content and were closely related to the first research question, while themes 4 and 5 encompassed broader reflections on personal and professional development as well as intentions to apply lessons learnt in future practice, predominantly addressing the second research question.

**Conclusions:**

Our findings demonstrate that reflective writing within our e-learning fostered students’ metacognitive engagement, enhanced their awareness of communication nuances, and supported transformative learning processes. Students not only recalled central course content but additionally explored topics such as dealing with their own uncertainty by examining their beliefs, attitudes, and challenges, recognising the role of communication in professional development. These findings align with previous research and highlight the potential of reflective writing to bridge theory and practice, contributing to students’ growth as empathetic and resilient healthcare professionals.

## Background

As medical education transitions from a transmissive, teacher-centred learning model to one emphasising reflective learning [1], inspired by constructivist learning theory [2], reflection has increasingly been integrated into curricula [3, 4]. Encouraging students to reflect on their own learning is held to be essential for cultivating a self-reflective attitude [5]. Reflection, a metacognitive process guiding future actions through deeper self-awareness and situational understanding [6], supports medical students in critically evaluating a situation and their own cognitive and behavioural responses. It allows them to identify new perspectives and lessons learnt, which they can then apply in future interactions [7, 8]. Through this process, learners not only gain cognitive clarity but also develop key interpersonal competencies that are essential in patient care. Reflection enhances empathy [9–12] and can enhance healthcare skills [13], particularly the quality of patient care [14]. Furthermore, studies highlight its impact on professional development [15], particularly in enhancing comfort with learning in complex situations, and deepening professional values [10]. Since reflection can foster self-knowledge [16], which has been shown to be one of the most important components of professional behaviour in medicine [17], it can thereby contribute to medical students’ professionalism [18–20]. Reflection is, therefore, widely regarded as a core competency for medical students [21].

In parallel to the shift from a teacher-centred to a reflective learning model, extending digitalisation represents another transformation in medical education [22], with e-learning offering a flexible approach for medical students to engage in individualised learning pathways [23]. Numerous studies have demonstrated the effectiveness of e-learning in fostering essential competencies of health care providers, including communication skills [24–27]. Despite being recognised as a promising teaching approach in medical education [28], e-learning requires careful consideration of its distinct characteristics, opportunities, and challenges. It often limits opportunities for immediate questioning, and personalised feedback is typically delayed. Moreover, instructors cannot directly monitor to students’ engagement and learning processes during the course. Effective interaction with content is known to facilitate learning. However, learner-content interaction can be particularly challenging for online learners, who must overcome learning difficulties independently [29]. Therefore, ensuring that students actively, thoroughly, and successfully engage with the material when learning autonomously is essential [30]. Such active engagement can be fostered through reflection, and research recommends that e-learning approaches should integrate methods that promote reflective practice [1, 30]. A widely used approach to facilitate self-reflection is reflective writing, which involves students composing texts based on their own experiences. This method has become well-established and valuable in medical education [9, 31–35]. Reflective writing enhances learning outcomes and positively influences students’ progress [36–40]. It brings key concepts to the forefront of students’ awareness [41] and provides an opportunity to make sense of internal processes, including successes, anxieties, and worries [42].

Synthesising these findings, it becomes evident that students, by reflecting on their experiences and internal processes, develop a deeper understanding of their personal and professional values, emotions, and reactions. This understanding, in turn, also play a vital role in developing clinical communication skills, which are essential for navigating complex clinical encounters with sensitivity and empathy [43]. Additionally, interactions between physicians and patients are a fundamental aspect of medical practice and thus a cornerstone of medical professionalism, representing one of the most critical aspects of medical practice [22, 44–46]. The quality of care is dependent on effective communication [47, 48], and, moreover, significantly affects the well-being of both patients [49, 50] and physicians [51], influencing physicians’ stress levels and potential for burnout [52]. To prepare for these challenges, medical students must develop clinical communicative competence [47, 53], going beyond theoretical knowledge or being able to reiterate phrases commonly used in interactions. They need to adapt their communication strategies in a context and situation-specific manner to the individual patient [54]. To develop these individual communication strategies, recent literature advises that communication training should encourage students to reflect [47] in particular on (observed or their own) experiences with patients [55].

Reflective writing can reveal students’ attitudes and challenges related to communication [56]. Implementing self-reflection in clinical communication training may increase students’ communicative competence [48], as reflection tends to correlate with communication skills [57]. However, self-reflection does not develop of its own accord as students progress through their education. Educational support is needed to help students understand approaches to self-reflection and its importance in enabling them to develop their abilities as well as to participate actively in reflective writing [58]. Gathering insights into learning processes facilitated through reflection is crucial in supporting students through their development, revealing learning bottlenecks or identifying where students may benefit from additional support to foster their learning [59–61].

While previous research suggests that analysing the content of students’ reflections can provide reliable insights into their learning experiences [62], there is still a gap in the existing literature, particularly regarding how medical students reflect on their learning within evolving educational settings that combine online learning and reflective practice. Although few studies have explored how online learning environments can aid students’ reflective practices [63] and might support them in reflecting on their learning experiences [64], the evidence remains limited. Little is known about how medical students perceive their learning experiences in online environments that embed reflective practice, especially in the context of clinical communication training. Thus, further research is needed to examine students’ reflective writings to gain deeper insights into their learning experiences in digitally mediated, reflection-oriented communication training.

Our study aims to address this gap and to contribute to the field by analysing medical students’ written reflections collected during an e-learning course on physician-patient communication. We explore how students perceived the course to gain insights into their learning experiences. We aimed to identify the learning content and topics students discussed, the challenges or benefits they encountered, revealed through their reflective writing. The study focuses on the following two research questions:

(I) What learning content do the students address in their reflections?
(II) What insights did the students gain through the course, and how did they perceive their learning?

## Methods

### Design and materials

We integrated repeated prompts for written reflection into a newly designed e-learning course on physician-patient communication and analysed the content of these reflective texts using thematic analysis. The e-learning course was developed to prepare first-year clinical students for a subsequent in-person teaching session, in which they independently conducted simulated patient interviews. The course consisted of three self-study 45-minute modules, each focusing on a specific topic: the basics of communication (Module 1), structuring consultations (Module 2), and empathy and emotions (Module 3). Each module combined theoretical content with quizzes and videos of simulated physician-patient interactions. These videos depicted (1) the initiation phase of a consultation (Module 1), (2) a complete patient interview in internal medicine (Module 2), and (3) a physician responding appropriately or inappropriately to patients’ emotional expressions (Module 3). At the end of each module, written reflections were consistently initiated in the same manner: students were prompted to engage in the reflection process by responding to guiding questions designed to deepen their understanding of the content and their learning experience [25, 30]. This approach aligns with recent literature recommending that reflective writing should be steered by written guidelines and open questions [59, 65]. Students provided with writing prompts use more metacognitive strategies compared to those without prompts [66], aligning with Dyment and O’Connell’s review, which emphasises the effectiveness of prompts for quality writing [59, 67]. Our reflection prompts were adapted from Koole et al. [8], focussing on the three core elements of reflection: awareness, understanding, and transfer (future application) [68–70]. Koole et al. used six guiding questions which made the three core elements of reflection visible and distinctly measurable, although they are usually merged within a reflection process. Unlike the original prompts by Koole et al., explicit questions about emotional responses were excluded to examine whether students would independently reflect on emotional aspects. The guiding questions were kept broad, allowing students to express their thoughts without being overly constrained [71]. We adapted the questions in the following way:

1. Reflection prompt: Awareness
  *“Describe some aspects that you noticed during the physician-patient interactions shown in the videos.”*
2. Reflection prompt: Understanding
  *“What did you learn? How? Why is that useful?”*
3. Reflection prompt: Impact on your future physician–patient interactions
  *“Which insights might be helpful for your future work? What do you plan for your physician-patient interactions?”*

We embedded these questions at the end of each of the three course modules. This approach reflects findings that multiple reflective writing enhances metacognitive awareness [59, 72], reduces resistance to reflection [59, 67], and provides a more accurate assessment of reflectivity than single samples [73]. The students had to answer the questions to proceed in the course. A minimum of 500 characters excluding spaces per prompt was specified to promote the reflection process. Assuming an average word length of six letters in informal German continuous text [74], this equates to 83 words. There was no time limit. Furthermore, the written reflections were not graded in order to reduce extrinsic motivations [75]. The study was approved by the Ethics Committee of the Faculty of Medicine at the Technical University of Munich and conducted in accordance with the Declaration of Helsinki. Participation was voluntary with written informed consent, and data were collected and analysed anonymously.

### Participants

The study was conducted during the winter semester 2020/21 at the Technical University of Munich. Participants included first-year clinical medicine students enrolled in a compulsory and self-study e-learning-course on physician-patient communication [25, 30]. Incorporating a mandatory course helped mitigate the potential bias of disproportionately motivated participants [76]. Prior to the course, students had not received training in reflective thinking. The way in which students engaged with the e-learning environment was flexible in terms of location and time.

In total, *N* = 121 students participated in the course, with *n* = 114 completing all modules and reflection prompts (80 female, 33 male, 1 other). The average age of participants was 22.02 years (SD = 2.48). Due to scope limitations and the considerable time investment required for qualitative thematic analysis, a random sample of reflections from 55 students, which represented the overall cohort’s demographics, approximately half of the total sample, with each student contributing nine reflections (three prompts per module across three modules), resulting in 495 reflections in total. This approach ensured a feasible yet sufficiently rich dataset, while maintaining methodological rigor.

### Analyses

We adopted thematic analyses after Braun and Clarke (2006) to identify students’ perceptions of their learning in the communication training. This approach, frequently used for reflective writing [77–79], allows an in-depth examination of the data to identify patterns and themes. Following the framework, the analysis involved six phases: (a) immersing in the data, (b) generating initial codes, (c) searching for themes, (d) reviewing themes, (e) defining and naming themes, and (f) producing the report. These phases are not linear but recursive, allowing researchers to return to previous stages, particularly when analysing complex data [77, 80]. To ensure the themes were derived directly from participants’ perspectives rather than predetermined frameworks, we employed an inductive approach. Specifically, we applied a codebook thematic analysis, which occupies a middle ground between coding reliability thematic analysis, and reflexive approaches to thematic analysis [81]. This codebook approach provides a structured framework for coding while allowing themes to emerge inductively through data engagement. Consensus between coders and intercoder reliability is not a measure of quality in this thematic analysis approach [82].

For analytical purposes, the three prompts written by one student within each module were analysed jointly to ensure that the reflection process was illustrated in its entirety while maintaining the visibility of the three core elements: awareness, understanding, and transfer. Two coders, the first author and a medical student research assistant, both analysed all 495 written reflections, with duplicate codes subsequently consolidated into a single code. On average, a text contained 266 words, with a slight decrease in text length across the three course modules (Module 1: 286 words; Module 2: 259 words; Module 3: 252 words). Both coders read the texts independently multiple times to ensure familiarity with the data and to develop a comprehensive understanding of the content. Coding was performed separately, with codes generated through a line-by-line analysis of the text. Codes were grouped into themes during the coding process. In regular joint meetings, the two coders discussed, condensed, and adjusted codes and themes. In addition, research team meetings with contributions from all co-authors were held to discuss the codebook and findings and to ensure consistency of approach. We used MAXQDA 2022 software for coding.

## Results

**Fig. 1:**
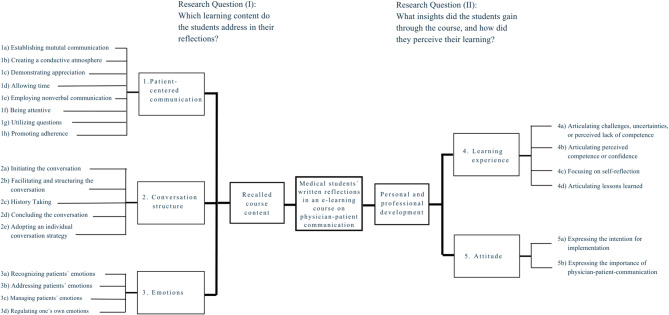
Mind Map *Focus of Themes*

Five main themes emerged from the data during the coding: 1. *Patient-centred communication*, 2. *Conversation structure*, 3. *Emotions*, 4. *Learning experience*, and 5. *Attitude.* The detailed analysis of the coded text segments within the themes revealed a distinction between Themes 1 to 3 and Themes 4 and 5, as illustrated in Fig. 1.

Themes 1 to 3 focus on specific learning content and contribute to answering the research question (I) *What learning content do the students address in their reflections?* We grouped these themes as *recalled course content*. In these themes, students mirrored the content of the three course models. In contrast, Themes 4 and 5 extend beyond specific content. In these themes, students discussed their insights on a more comprehensive level instead of specifically and in relation to concrete content: text segments of Themes 4 and 5 primarily include more critical reflections on students’ own learning and competencies, concrete intentions for their future conversations with patients, and an awareness of the significance of physician-patient communication, both for patients and for themselves as physicians. We therefore examined Theme 4 *Learning Experience*, and Theme 5 *Attitude*, in more detail to explore research question (II) *What insights did the students gain through the course*,* and how did they perceive their learning?* We categorised Themes 4 and 5 as reflections on consequences for *personal and professional development*. In the following sections, we provide examples of all themes, highlighting patterns and recurring topics. Firstly, we present an overview of all five themes. Subsequently, we deep dive into Themes 4 and 5 since they proved particularly interesting regarding personal and professional development.

The five identified themes and their key insights are summarised in Table 1.

**Table 1:** Themes, Key Aspects, Codes, and Code Frequency

Theme	Key Aspects	Code	Code Frequency
1. Patient-centred communication	building trust; creating a welcoming and empathetic environment; using nonverbal cues to enhance the conversation; promoting mutual communication, shared understanding, and adherence	1b) Creating a conducive atmosphere	177
		1c) Demonstrating appreciation	132
		1 h) Promoting adherence	109
		1 d) Allowing time	105
		1a) Establishing mutual communication	103
		1e) Employing nonverbal communication	102
		1 g) Utilising questions	84
		1f) Being attentive	59
		code frequency for theme 1	**871**
2. Conversation structure	successful beginnings of conversations; managing smooth transitions between stages; the importance of summaries for shared understanding	2b) Facilitating and structuring the conversation	240
		2a) Initiating the conversation	161
		2c) History taking	42
		2 d) Concluding the conversation	38
		2e) Adopting an individualised conversation strategy	29
		code frequency for theme 2	**510**
3. Emotions	recognising and responding to patients’ emotions; managing emotional challenges; developing emotional resilience; recognising the role of emotions	3c) Managing patients’ emotions	203
		3a) Recognising patients’ emotions	79
		3b) Addressing patients’ emotions	60
		3 d) Regulating one’s own emotions	34
		code frequency for theme 3	**376**
4. Learning experience	identifying lessons learnt, skill gaps, or perceived support through the course, engaging in deep self-reflection, resulting in new understanding and perspectives	4 d) Articulating lessons learnt	338
		4a) Articulating challenges, uncertainties, or perceived lack of competence	28
		4c) Focusing on self-reflection	20
		4b) Articulating perceived competence or confidence	13
		code frequency for theme 4	**399**
5. Attitude	committing to applying learnt strategies; acknowledging communication as a cornerstone of patient care	5a) Expressing the intention to implement	275
		5b) Expressing the importance of physician-patient-communication	35
		code frequency for theme 5	**310**
		code frequency in total	**2**,**466**

We coded a total of 2,466 text segments belonging to the five emerged themes (see Table 1). These themes can be summarised as follows:

- Theme 1 *Patient-centred Communication* highlights the importance of fostering an empathetic and supportive environment during interactions with patients. Students emphasised strategies such as establishing mutual communication, creating a conducive atmosphere, and demonstrating appreciation of patients. Additionally, they recognised the need to allocate sufficient time for patient concerns and to utilise both verbal and nonverbal cues to enhance attentiveness. In addition, students mentioned the impact of tailored questions and patient adherence.
- Theme 2 *Conversation Structure* focuses on the essential elements of structuring conversations. Students discussed strategies for successful beginnings and highlighted the significance of smooth transitions between conversation phases to ensure clarity for patients and physicians. They recognised the value of structured approaches to history-taking, such as the Calgary-Cambridge Guide [83], to ensure comprehensiveness. Additionally, students underscored the importance of concluding conversations with summaries and providing opportunities for patients’ questions. They also stressed the need to develop personalised strategies to tailor their communication style to individual patients’ needs.
- Theme 3 *Emotions* underscores the significance of addressing both the patient’s and physician’s emotions. Students recognised the importance of identifying and explicitly addressing patients’ emotions to build trust and to strengthen the physician-patient relationship. Additionally, they highlighted the need for managing patient emotions with empathy, without amplifying fears, while providing reassurance. Reflecting on their own emotional regulation, students acknowledged the impact of their personal feelings on interactions and expressed a commitment to self-awareness to avoid countertransference.
- Theme 4 *Learning Experience* focuses on the new insights, challenges, and opportunities students identified while engaging with the learning material. In addition, Theme 4 covers text elements of intense self-reflection, enabling perspective change and transformative thinking leading to new understanding. Students evaluated their knowledge and articulated specific lessons learnt, such as the importance of small yet fundamental elements such as proper introductions and their impact on building strong physician-patient relationships. A challenge that they frequently named was the difficulty of balancing empathy with avoiding the amplification of emotions, especially in early practice when techniques are not yet fully internalised. While many students expressed uncertainty about their abilities, others reported a growing sense of competence, bolstered by practical guidelines and theoretical knowledge.
- Theme 5 *Attitude* examines students’ focus on applying their lessons learnt in future practice and their recognition of communication’s crucial role in physician-patient interactions. Many students emphasised the value of starting conversations with open questions and adapting their verbal and nonverbal communication to patient needs. They reflected on the need for a respectful and unbiased approach, particularly during initial diagnoses, and noted the intention to be aware of patients’ and their own emotions. Additionally, students expressed a deeper awareness of how impactful communication is, not only for fostering trust but also for ensuring that patients follow medical recommendations.

### Code distribution

The codes from Theme 1 *Patient-centred Communication* were applied most frequently. These codes were predominantly mentioned in the reflective texts collected during the first course module. As described above, these reflective texts were, on average, slightly longer than those from subsequent modules. Module 1 introduces the fundamentals of patient-centred communication, covering a wide range of specific aspects reflected in the codes of Theme 1. Students frequently referred to these aspects as insights. While the second module, which focuses on the phases of conversations, primarily utilised codes from Theme 2 *Conversation structure*, codes from Theme 1 also appeared prominently. Students frequently revisited topics from Theme 1 in Module 2, such as creating a positive atmosphere, active listening, and guiding conversations with questions. Module 3 primarily focuses on managing emotions. Here, codes from Theme 3 *Emotions* were most frequently applied. Themes 4 *Learning Experience* and 5 *Attitude*, by contrast, appeared almost evenly distributed across reflective texts from all modules.

Since our analysis focused less on code frequency and more on the themes, key insights, and potential patterns, we particularly analysed the text segments associated with each code in detail. Table 2 provides three examples of coded text segments for each code. The quotes were originally written in German and have been translated as faithfully as possible to the original, without linguistic improvements or modifications of meaning.

**Table 2:** Codes and representative quotes

Code	Representative Quote
Theme 1: Patient-centred Communication	
1a) Establishing mutual communication	“What stood out to me in this example was the aspect of patient ‘empowerment’.”/“Using ‘we’ language and making sure the patient agrees is crucial. It’s not just about me being satisfied with the outcome, but (especially) about the patient feeling the same way.”/“I want to meet the patient more as an equal during the conversation.”
1b) Creating a conducive atmosphere	“I’ll make an effort to take my time and, even when there’s little time, create a calm environment where patients feel comfortable expressing their concerns.”/“Having a pleasant conversation atmosphere is crucial, ensuring the patient leaves the discussion feeling positive.”/“Setting shared goals for the conversation, addressing the expectations and views of both physician and patient, fosters a good atmosphere and mutual satisfaction.”
1c) Demonstrating appreciation	“Patients need to feel valued and respected to develop a strong relationship with their physician, which contributes to the success of their treatment.”/“In future conversations, I aim to show more appreciation and compassion toward patients, approaching discussions more openly and empathetically.”/“A physician’s physical positioning toward the patient can also make them feel more valued, which is important for a good physician-patient relationship.”
1 d) Allowing time	“I learnt the importance of letting patients speak first before steering the conversation with targeted questions.”/“No matter how much time pressure or stress there is, I want to always be friendly and never make patients feel rushed.”/“I want to give the patient the feeling that I take their complaints seriously and take time to address them as a person, instead of just dealing with them quickly to get on with examinations and diagnosis.”
1e) Employing nonverbal communication	“Nonverbal communication is key, but paraverbal cues, such as speech speed and tone, can also convey attention, appreciation, and interest.”/“The physician’s introduction, body language, tone, and eye contact played a significant role.”/“I observed how the patient’s nonverbal behaviour shifted depending on how the physician interacted with them.”
1f) Being attentive	“The physician gave the patient her full attention, doing nothing else, ensuring nothing was missed.”/“Sometimes, the problems aren’t obvious, so paying attention to what the patient says and how they behave, and asking follow-up questions when needed, is crucial for understanding the full picture.”/“Active listening and paraphrasing show the patient that you’re focused on them, taking their concerns and fears seriously.”
1 g) Utilising questions	“I think it’s great to start a conversation with an open-ended question to understand the reason for the visit. Afterwards, I would ask targeted questions to learn more.”/“I’ve learnt when to use open-ended, closed, or targeted questions. For instance, I plan to start with open-ended questions and avoid leading questions.”/“Over time, questions should become more focused to manage time effectively.”
1 h) Promoting adherence	“As mentioned earlier, I think it’s very beneficial to define the purpose and goals of the conversation upfront and conclude by ensuring the patient understands what was discussed.”/“The physician explains their reasoning to the patient, helping them understand the diagnosis and treatment plan, and enabling them to participate in the next steps.”/“I also plan to involve patients in treatment decisions as much as possible, tailoring them to the patient’s needs and fostering a balanced dialogue.”
Theme 2: Conversation Structure	
2a) Initiating the conversation	“Introducing yourself and waiting until the patient is seated before starting the medical history is essential.”/“First impressions significantly affect the conversation’s progress and shouldn’t be underestimated.”/“At the beginning, it’s important to set the content and goals of the discussion and explain the flow of the conversation.”
2b) Facilitating and structuring the conversation	“I plan to pause briefly during transitions in conversations to recap key points and identify what else I need to know. I’ll then communicate the purpose of my next questions to the patient.”/“It was nice to see how effective seemingly simple things like summarising what was just said or clearly transitioning verbally to a new part of the conversation can be, to not lose track myself but also to ensure the patient’s attention and understanding.”/“I noticed that the physician provided the patient with a clear framework, so they always knew where they were in the conversation.”
2c) History Taking	“Using a guide for medical history-taking ensures no important details are overlooked. Applying the Calgary-Cambridge Guide gives the conversation structure and keeps it on track.”/“In today’s module, I learnt which important aspects need to be considered and worked on during the medical history taking so that you can reach an accurate initial diagnosis within a reasonable timeframe.”/“The patient appreciated a welcoming, distraction-free setting during the medical history, as it helped them feel more comfortable in an unfamiliar situation.”
2 d) Concluding the conversation	“After the physical exam and at the end of the visit, everything was summarised, and the patient was given the chance to ask questions.”/“I will also try to conduct the end of conversations in my future work with shared decision-making, summarising, and clarifying open questions.”/“Summarising the patient’s statements reassures them that you’ve understood, and explaining the next steps is equally important.”
2e) Adopting an individualised conversation strategy	“Before conversations, I plan to quickly review my approach or bring notes to follow a proper sequence and avoid missing anything.”/“For future conversations, I plan to create a mental roadmap beforehand to ensure the discussion is well-structured and effective.”/“Over time, I want to develop my own method for conducting patient interviews.”
Theme 3: Emotions	
3a) Recognising patients’ emotions	“I learnt how important it is for physicians to recognise patient emotions.”/“I want to practice recognising emotions and dealing with them correctly in private conversations and then apply these techniques in patient discussions.”/“Patients often bring strong emotions and fears to the conversation, so it’s vital to acknowledge them.”
3b) Addressing patients’ emotions	“I’ve learnt that emotions are a key part of physician-patient conversations and should be addressed explicitly using models like NURSE. Expressing understanding is crucial.”/“Naming and addressing emotions directly shows empathy and strengthens the physician-patient relationship.”/“Very often, patients feel too inhibited or have too high a threshold to communicate their true emotions. That’s why it’s especially important for physicians to break the taboo and directly address those feelings.”
3c) Managing patients’ emotions	“It was notable that the physician took the patient’s concerns seriously without dramatising them, striking a balance between acknowledging fears and not amplifying them.”/“Helping patients through emotional struggles like hopelessness, despair, and lack of motivation is more impactful than one might think. Offering help and showing understanding is vital.”/“I learnt that the more anxious or stressed the patient appears, the calmer the physician needs to remain while also reassuring the patient.”
3 d) Regulating one’s own emotions	“I’ve become more aware of how important my own emotions are, which I unconsciously bring into conversations and even into my interactions with others, ultimately influencing the dynamics.“”/“I will try my best to empathise with my patients’ emotional lives so I can better understand their perspective. Additionally, I want to pay closer attention to my own emotions and name them to avoid any countertransference on my part.”/“I also plan to pay attention to my own feelings and to what the patient triggers in me.”
Theme 4: Learning Experience	
4a) Articulating challenges, uncertainties, or perceived lack of competence	“It’s really not easy to strike the right balance between acknowledging emotions on one hand and unintentionally amplifying emotions, like fear, on the other hand.”/“I imagine that actually implementing everything you plan in a medical conversation is significantly more challenging.”/“However, my concern is that, especially in the beginning when these strategies aren’t fully internalised yet, conversations might come across as very technical.”
4b) Articulating perceived competence or confidence	“I was reminded that the physician-patient conversation is a key part of the profession and how much can go wrong, but also that there are clear and easy-to-remember guidelines to create a good start to any conversation.”/“With the theoretical background I’ve learnt, I think I can find a good balance in dealing with emotions during conversations.”/“I would say about myself that I am generally a very approachable person and have no trouble making a friendly impression, but it is very helpful to hear specific examples like greetings once, so you have a certain tool you can use in specific situations.”
4c) Focusing on self-reflection	“It’s important to reflect on yourself every now and then: How would I have felt in this conversation? Would I have felt well taken care of? At the same time, you need to keep in mind that every person is different.”/“Before working on this module, I thought you could make these kinds of judgments pretty well using intuition alone. It turns out I was wrong.”/“It’s not just about paying attention to the patient but also to yourself. You should ask yourself what emotions the conversation brings up in you, how you interpret the patient’s statements, and whether your interpretation is fair.”
4 d) Articulating lessons learnt	“What stood out to me the most was how important it is to introduce yourself properly.”/“After this module, I can definitely say that I will put more focus on patient conversations in my future career.”/“For me, it became especially clear how important some basic elements of communication and interaction are, not only for a successful physician-patient conversation but also for building a strong relationship between the two.”
Theme 5: Attitude	
5a) Expressing the intention to implement	“This technique of starting with open questions and becoming more specific will definitely be something I try out, as it is even more effective in normal conversations due to its simplicity.”/“For my future physician-patient conversations, I aim to use appropriate gestures and facial expressions myself and to recognise and respond well to the patient’s nonverbal signals.”/“For my future work as a physician, I plan to place great value on a respectful and warm start to a patient conversation and not to be too biased if I already have a suspicion about a possible diagnosis.”
5b) Expressing the importance of physician-patient-communication	“I now realise how important communication with the patient is, not just so that patients find me likable, but also to get more information from them on a professional level and to ensure that they trust me enough to follow my suggestions.”/“After this module, I can definitely say that I will put more focus on patient conversations in my future career.”/“I also understand now how important physician-patient communication is, as it happens multiple times a day and is, alongside medical knowledge, essential to master in order to become a good physician.”

### Extended reflection beyond specific content in themes 4 and 5

While Themes 1 to 3 focus on specific learning content, Themes 4 and 5 reflect a broader level of engagement and address personal and professional development. They include more comprehensive reflections, such as critical engagement with one’s own learning processes, perceived competencies, intentions for future physician-patient interactions, and an awareness of the significance of physician-patient communication. The following sections present illustrative examples, highlighting recurring patterns within these reflections.

### Theme 4 learning experience

The following section summarises recurring themes and patterns from students’ written reflections categorised under Theme 4, *Learning Experience*. This theme encompasses students’ perceptions of communicative challenges and uncertainties (Code 4a *Articulating challenges*,* uncertainties*,* or perceived lack of competence*), their occasional expressions of confidence (Code 4b *Articulating perceived competence or confidence*), their engagement with self-reflection as a learning strategy (Code 4c *Focusing on self-reflection*), and the concrete lessons they identified from the learning experience (Code 4 d *Articulating lessons learnt*). Overall, students’ reflections highlight a strong awareness of the complexities of clinical communication, with a predominant focus on challenges and areas for growth rather than perceived competence. While uncertainties regarding conversational flow, structuring, and emotional engagement were frequently articulated, students also demonstrated active engagement in self-reflection and a keen recognition of key insights, underscoring the value of ongoing reflective practice in developing communication skills.

#### 4a articulating challenges, uncertainties, or perceived lack of competence

Students most frequently addressed challenges in managing the structural aspects of patient communication. A central difficulty was maintaining a smooth conversational flow: “*For me personally*,* it would be important to learn how I can stay relaxed during a conversation and not feel like I am forgetting to ask lots of important questions during the medical history*.” Another common concern was transitioning between topics without disrupting the natural rhythm of the conversation: “*I especially appreciated that the video clearly showed how to transition smoothly from one topic to another*,* as this is something I’ve always found challenging.*” Another student stated: “*Personally*,* I always find it difficult to create seamless transitions without abrupt breaks in the flow of the conversation*.” Moreover, some students noted a tendency to adopt overly factual communication styles: “*I think I tend to make conversations overly informative and factual rather than focusing on the patient.*” Another student recognised the importance of balancing structure with empathy, stating: “*‘I noticed how the physicians in the videos struck a good balance between following structured approaches and demonstrating genuine empathy. It never felt like they were rigidly checking off points; instead*,* the conversations in the positive examples always seemed very natural.*” Regarding the transition to practice, some reflections highlighted concerns about applying theoretical knowledge in practical settings: “*I’ve also learnt theoretically about which types of questions tend to be asked at which stages of a conversation (starting with open questions*,* then moving to targeted and suggestive ones) to get the most information in a limited amount of time. However*,* I doubt that I’ll be able to put this into practice easily right away*.”

#### 4b articulating perceived competence or confidence

While analysing the texts, we noted that in contrast to the repeatedly mentioned uncertainties regarding personal competence (Code 4a), perceived certainty and competence (Code 4b) were rarely highlighted and infrequently coded. We identified a few comments indicating a perceived increase in confidence: *“By becoming familiar with a specific conversation technique beforehand*,* I can gather information more effectively*,* and that this leads to better understanding*,* not just on my side*,* but also for the patient.”* Or, as another student expresses it: *“I think I tend to keep conversations very factual. With tools like WWSZ*,* I feel like I can improve this — build a better connection with patients and make the conversations more comfortable for myself*,* too.”* However, the students addressed previously acquired competencies less frequently and instead reflected more extensively on insights gained specifically from the e-learning course and on lessons learnt (Code 4 d). Due to the limited data, we cannot draw conclusions about Code 4b.

#### 4c focusing on self-reflection

Self-reflection was visible, with students emphasising its importance for enhancing communication and emotional awareness: “*Since I’m naturally one of those people who tend to be ‘too empathetic’ rather than not empathetic enough*,* I often don’t dare to address what I observe in others because I’m afraid they might find it too personal or intrusive*.” Additionally, several students highlighted the significance of recognising and managing their own emotions during patient interactions: “*Also — and I think this is something I still have a lot to learn — I plan to become more aware of my own emotions when I go into a conversation or as they arise during the conversation. This way*,* I can avoid falling into the trap of negative countertransference.*” Students also repeatedly acknowledged the value of regularly reflecting on themselves to refine their approach: “*I plan to regularly question myself*.” Furthermore, reflections on seemingly minor details, such as self-introduction during greetings, illustrated students’ attention to patient perspectives: “*In the video examples where the greeting didn’t include a self-introduction*,* it would have felt strange to me as the patient*.”

#### 4d articulating lessons learnt

Lessons learnt about beginning and structuring conversations as well as about dealing with emotions emerged as the two most common, recurring topics. A frequently mentioned aspect was the importance of giving patients the opportunity to express themselves fully, particularly at the beginning of a conversation: “*What sticks with me most is that*,* at the start of the conversation*,* you should give the patient space to share their story*,* but then also ask targeted questions to get the information you need*.” Another student wrote: “*I think it’s especially important to give the patient space and time for their questions*,* to avoid interrupting them too quickly*,* but also to step in gently when necessary to keep the conversation balanced*.” In addition, students frequently highlighted aspects related to emotions as their lessons learnt. They noted the value of empathy as a two-way process that benefits both the physician and the patient: “*On the one hand*,* I found it very impressive that empathy is not just about recognising emotions or putting yourself in someone else’s shoes*,* but that mutual empathy can benefit both the physician and the patient*.” Additionally, emotional nuances in communication were frequently mentioned: “*I didn’t realise that emotions often come through indirectly*,* hidden behind other questions*.” Furthermore, students reflected on various aspects of the complexity of communication: “*What I found surprising in the video was how much was communicated*,* both verbally and nonverbally*,* in such a short time.*” They recognised the significance of seemingly minor details in shaping communication outcomes: “*I was very surprised by how much of a difference small details in communication can make.*” Additionally, students acknowledged the challenges of mastering communication skills: “*I learnt that many mistakes can be made in clinical communication*,* and it’s not as easy as one might think. On the other hand*,* it’s good that we are now learning what matters so that we can spot mistakes more easily and make fewer of them in the future.”*

### Theme 5 attitude

The following section presents recurring patterns and key topics from student reflections categorised under Theme 5, *Attitude*, describing students’ intentions to implement their lessons learnt in practice (Code 5a *Expressing the intention to implement)* and their awareness of the importance of communication as expressed in their written reflections (Code 5b *Expressing of the importance of physician-patient communication).*

#### 5a expressing the intention to implement

Regarding the intention to implement, the topics of structuring a conversation and emotions were the most frequently mentioned, mirroring the articulated lessons learnt. Students repeatedly expressed their intentions to approach patient conversations with greater structure and clarity, reflecting specifically on how to begin a conversation: “*In the future*,* I will try to think about a good conversation start*,* which means not only defining the goal and intent but also setting a time frame for myself and communicating it to the patient.*” The desire to create a safe and open environment for patients was a common topic: “*I’ll make an effort to give patients the space to talk about their psychosocial situation*,* try not to interrupt them*,* and follow the rules for verbal*,* non-verbal*,* and para-verbal communication so they feel that someone is truly listening to them*.” Several reflections focused on achieving a balance between guiding conversations and allowing patients to express themselves freely: “*I plan to pay closer attention to the balance between ‘letting the patient speak without interruption’ and ‘guiding the conversation in a certain direction’ to find the middle ground between these two extremes*.” Moreover, students frequently highlighted the importance of summarising and verifying patient information: “*I plan to better assess the knowledge level and understanding of the conversation by summarizing and asking follow-up questions.*” Another student wrote: “*Lastly*,* I plan to summarise what the patient said at appropriate points in the conversation to develop a shared understanding of the symptoms and treatment*,* and to provide space for possible questions*.” Additionally, students also emphasised the need for structured communication strategies: “*I especially plan to have a certain framework ready before every conversation*,* if possible*,* to work effectively and not miss or forget information*.” Another stated: “*For future patient conversations*,* I plan to start with open questions and make them more precise as the conversation progresses*,* to follow an initial suspicion and gather the exact information I need.*” Besides conversation structure, emotions were the central topic. Empathy and emotional awareness were central to students’ planned approaches: “*I also plan to address and respect the patients’ emotions*,* approach them empathetically*,* and support them this way*,* for example*,* by reducing potential fears through communication and joint treatment planning.*” Another student mentioned the importance of avoiding phrases that might unintentionally amplify a patient’s fears: “*I will also try to avoid phrases like ‘your fear is justified*,* but…’ since patients*,* especially those who might see the physician more as an authority figure*,* could become even more scared. Instead*,* I would try to use phrases like ‘I can understand your uncertainty*’.” Students repeatedly articulated their commitment to paying closer attention to emotions: “*Handling the patient’s feelings appropriately can significantly influence their satisfaction. Therefore*,* I aim to better understand and address these emotions more often*,* to engage with them and work out the reasons for them with the patient*.”

#### 5b Expressing the importance of physician-patient communication

Students recognised the essential role of communication in fostering successful physician-patient relationships: “*Good communication is essential for building a strong physician-patient relationship*.” Another student wrote: “*A good start to a conversation is*,* of course*,* nothing new*,* but this module really made me realise how important it actually is*.” A further reflection expressed the impact of initial impressions with the words: “*If the start of the conversation doesn’t go well*,* or if the patient doesn’t feel comfortable or taken seriously*,* it not only makes the diagnosis and treatment harder but also creates unpleasant feelings for both the patient and the physician*.” The impact of physician-patient communication on both sides, patient as well as physician, was noted by several students. They pointed out the benefits of good physician-patient-communication, addressing both physical and mental health concerns: “*For my future work as a physician*,* I take away that a good and effective physician-patient conversation is not just about helping the patient with their physical problems but also with the mental struggles that come with it*,* like hopelessness*,* despair*,* or lack of motivation*.” Another stated: “*I’ve learnt that a good and balanced physician-patient relationship benefits both sides*,* the physician and the patient. It increases overall satisfaction*,* improves the patient’s compliance*,* reduces the physician’s stress*,* and helps the patient feel less anxious or insecure*.” Similarly, another student stated: “*I believe that good communication in medicine is very important and benefits both sides. That’s why it’s worth reflecting on your communication skills every now and then and striving to get better*.”

Another recurring topic was the relationship between communication and diagnostic accuracy: “*Physician-patient conversation plays a very important role in everyday medical practice. It’s not just about building a trusting relationship as a foundation for collaboration in terms of shared decision making. In fact*,* 50% of diagnoses are already made during the conversation*.” Similarly, the success of treatment was often linked to effective communication: “*The success of a treatment depends on the relationship and communication with the patient. Only with good communication will the patient follow the physician’s advice and recommendations*.” Overall, students noticed the broader implications of communication for everyday medical practice: “*Since physician-patient conversations are among the most common tasks in medicine and are crucial for both the patient’s and the physician’s satisfaction*,* they are undoubtedly very useful in everyday medical practice*.”

## Discussion

This study responds to the call in the literature for a deeper understanding of how medical students perceive their learning experience in an e-learning course on communication skills that fosters reflection, identifying the learning content and topics students discussed, and the challenges or benefits they stated, revealed through their reflective writing. The conducted thematic analysis provided valuable findings and insights into students’ learning process and how they perceived their learning. The following sections discuss these findings, particularly key aspects, patterns, and relations to previous literature.

Regarding the first research Question 1 “*What learning content do the students address in their reflections?*” the findings revealed that students addressed all key topics conveyed in the course. Theme 1 *Patient-centred Communication* was particularly prominent in Module 1, where foundational principles of patient-centred communication were introduced. Students emphasised the importance of building trust, employing nonverbal communication, and fostering mutual understanding, which underscores the significance of these skills in creating successful and collaborative physician-patient relationships [47]. Theme 2 *Conversation Structure* and Theme 3 *Emotions*, which focused on conversation structure and emotional dynamics, respectively, were most evident in Modules 2 and 3.

While the reflections belonging to Themes 1–3 showed that students internalised the intended course material, Themes 4 *Learning Experience* and 5 *Attitude* extended beyond recalled course content and related directly to the second research question: *What insights did the students gain through the course*,* and how did they perceive their learning?* These two themes were more evenly distributed across all modules and revealed deeper insights into students’ personal and professional development, including lessons learnt, challenges, skills gaps, and intentions for application in practice (see Fig. 1, Mind Map). They addressed topics not explicitly covered in the course design, such as the experience and management of uncertainty. Through metacognitive engagement, students uncovered aspects that were not part of the formal learning objectives, as, for instance, becoming aware of one’s own uncertainties was not a targeted learning goal of the course.

Summarising Themes 4 and 5, students frequently identified challenges in maintaining conversational flow, balancing structure and empathy, and translating theoretical knowledge into practice, which they recognised as critical areas for improvement (Code 4a *Articulating challenges*,* uncertainties*,* or perceived lack of competence*). Uncertainty was a frequently recurring issue, and one which has also been described in previous research on reflective writing in medical education. It has been studied from various perspectives in medical education, but less frequently with a specific focus on reflective writing [71, 84]. Aligning with previous research, uncertainty was mentioned in relation to other topics in our study, not as a separate theme [71]. Nevalainen et al. draw the connection to professional growth, stating that reflective writing can help students to express and cope with uncertainty, supporting professional development [84]. The connection that professional development is enhanced through reflective writing is well-established and frequently evidenced in academic discourse [15, 18].

Reflections on seemingly minor details, such as self-introduction during greetings (Code 4c *Focusing on self-reflection)*, mirror students’ growing awareness of the subtle nuances that shape patient trust and comfort, and are consistent with previous research demonstrating that reflection enhances situational awareness, awareness of others, and self-awareness [85]. Students’ reflections highlighted the complexity of communication, emphasising the significance of small details, the balance between listening and guiding conversations as key insights, and the importance of dealing with sometimes hidden emotions (Code 4 d *Articulating lessons learnt*). Emotional nuances were frequently noted. This recognition of subtle emotional cues reflects a deeper understanding of patient communication dynamics. Students acknowledged the importance of managing their own as well as patients’ emotional needs, highlighting the reciprocal benefits of empathy for both physician and patient [10]. This demonstrates a nuanced understanding of the emotional aspects of communication. It indicates how self-reflection might support the development of emotional resilience and professionalism, critical attributes for mitigating physician burnout [52].

Students also articulated intentions to integrate specific communication strategies into their clinical work, including appreciative beginnings of interactions with patients, developing their own routines to structure conversations, and addressing emotions during conversations (Code 5a *Expressing the intention to implement*). This reflects the transformative potential of reflective writing in fostering self-awareness and bridging the gap between theory and practice [3, 73]. Finally, students underscored the vital role of effective communication in diagnosis, treatment success, and overall satisfaction for both patients and physicians, recognising its centrality in everyday medical practice (Code 5b *Expressing the importance of physician-patient-communication*). This illustrates students’ recognition of the holistic nature of patient care. Their reflections further indicate an understanding of how communication fosters professional well-being alongside patient care, emphasising the mutual benefits communication.

In summary, our findings support previous studies stating the crucial role of reflective writing in fostering reasoning skills [42]. In particular, aligning with previous research, our findings highlight that students’ reflections seem to make them aware of their beliefs, feelings, challenges, and attitudes [38, 86, 87], and provided an opportunity to understand internal processes including successes, anxieties, and worries [42]. Most notably, the text segments related to Codes 4c *Focusing on self-reflection*, and 4 d *Articulating learning* mirrored the discovery of faults in what they previously perceived to be right, thus demonstrating transformative learning leading to new insights and understanding, as previously described by other researchers [86, 88]. The students openly reflected on their misjudgements in their texts, supporting previous research findings that reflective exercises provide students with a safe learning environment to engage with the complex topic of patient interactions [21, 89]. Overall, the five identified themes provide comprehensive answers to our research questions, with Themes 1 to 3 addressing recalled course content and respond the first research question, while Themes 4 and 5 address the second research question, highlighting students’ metacognitive engagement, transformative learning, and professional development.

### Limitations and future studies

While this study provides valuable insights into the reflective learning processes of medical students, several limitations should be considered.

Firstly, among the acknowledged risks in qualitative research are lack of interpretative transparency and the potential for overgeneralisation [18]. To address this, we aimed for a high degree of transparency in our study design, including a detailed description of the course, the methodology, and the analytic process. This transparency allows readers to critically assess the findings and mitigates the potential biases inherent in qualitative analyses.

Secondly, fatigue effects must be considered due to the repetitive reflection prompts. The reflection prompts were provided with the same wording across all course modules to ensure comparability and because previous research has shown single samples to be less suitable for accurate reflectivity assessments [73]. Nevertheless, students might have been less motivated to compose written reflections towards the end of the course, which would explain the slightly decreasing text lengths over the course.

Thirdly, we did not teach students how to reflect in advance. However, teaching students the basics and importance of reflection before the reflection exercise seems to improve their ability to reflect [3, 90]. Explicitly communicating the purpose and expectations of reflective writing to students may reduce resistance and enhance the extent or quality of reflections. As prior research highlights, making the reflective process’ value clear to students can improve engagement [67]. In most studies, participants were trained in advance [42]. We did not pre-train students in this study, nor did we teach them the importance of reflection, as this might have distorted the findings. We intend to include pre-training on reflective practice in future implementations to support students in engaging more deeply with the prompts. We also did not provide feedback to our students, as it might have influenced their motivation and the results of the study. However, there is broad evidence that ongoing feedback can improve reflection [3, 91, 92], and we plan to incorporate it into our subsequent courses.

Another limitation of this study concerns the linguistic and cultural background of the participants. As the degree program in Medicine at the Technical University of Munich is taught entirely in German, the participants were students from Germany and native speakers of German. Their texts were translated into English for analysis, with the translation carried out as literally as possible to preserve the original meaning and style. Consequently, potential influences of multilingual or multicultural backgrounds on communication could not be examined.

Lastly, future research will have to clarify the development of reflective skills over time and demonstrate whether and how increased reflective capacity leads to better physician-patient interactions. There is limited research on the relationship between the quality of reflections and the academic achievement of medical students [62], and previous studies have shown mixed results [93], or reported little evidence on how reflection correlates with other measures or performances in medical school [62, 94].

Despite these limitations, our findings underscore the potential of reflective writing to foster metacognitive awareness and awareness of aspects of clinical communication. This contributes to a growing body of evidence supporting the value of reflective writing in medical education. Future studies could build on these findings to explore longitudinal effects and to investigate the relationship between reflective capacity and clinical performance. Our study also provides opportunities for practical implementation. Embedding structured reflective prompts within e-learning modules enables students to engage deeply with the course content and express meaningful insights. These reflection prompts are easily embeddable into various digital learning management systems.

## Conclusion

In conclusion, this study demonstrates the value of reflective writing in fostering new understanding, essential communication skills, and supporting students’ personal and professional development in medical education. By aligning course content with structured reflection, our transferable e-learning approach effectively engaged students in achieving key insights, identifying personal challenges or competencies, committing to future implementation of their lessons learnt in practice, and becoming aware of the importance of communication while interacting with patients. Our findings clearly evidenced and illustrated the value of reflection in enhancing students’ ability to achieve new insights, refine professional values, and engage in transformative learning. This was neatly expressed by one student while reflecting on when and how to initiate different phases of a conversation: “*Before working on this module*,* I thought you could make these kinds of judgments pretty well through intuition alone. It turns out I was wrong.”*

## Data Availability

The datasets used and/or analysed during the current study are available from the corresponding author upon reasonable request.
